# The Impacts of Positive Psychology on Depression, Anxiety, and Conflict Resolution Tactics in Pregnant Women Experiencing Intimate Partner Violence During the COVID‐19 Pandemic: A Quasi‐Experimental Study

**DOI:** 10.1002/hsr2.72214

**Published:** 2026-04-28

**Authors:** Sohaila Ordikhani, Sousan Heydarpour, Nasrin Jaberghaderi, Nader Salari

**Affiliations:** ^1^ Student Research Committee Kermanshah University of Medical Sciences Kermanshah Iran; ^2^ Department of Reproductive Health, School of Nursing and Midwifery Kermanshah University of Medical Sciences Kermanshah Iran; ^3^ Department of Psychiatry, School of Medicine Kermanshah University of Medical Sciences Kermanshah Iran; ^4^ Department of Biostatistics, School of Health Kermanshah University of Medical Sciences Kermanshah Iran

**Keywords:** anxiety, conflict resolution strategies, COVID‐19, depression, intimate partner violence, positive psychology, pregnant women

## Abstract

**Background and Aims:**

Intimate partner violence (IPV) can negatively impact both maternal and fetal outcomes. The present study aimed to determine the effects of positive psychology‐based interventions on the levels of depression and anxiety symptoms, as well as conflict resolution strategies, in pregnant women exposed to IPV during the COVID‐19 pandemic.

**Methods:**

A quasi‐experimental design was employed involving 74 pregnant women with a history of IPV. Participants were assigned to either an intervention group (*n* = 37) or a control group (*n* = 37) through block randomization. The intervention consisted of 8 weekly sessions of a structured positive psychology program emphasizing positive emotional experiences, adaptive coping, gratitude practices, contentment exercises, and identification of personal strengths. The control group received routine prenatal care. IPV exposure was assessed using a domestic violence screening checklist. Outcomes were measured using the Conflict Tactics Scales, the Spielberger State–Trait Anxiety Inventory, and the Edinburgh Postnatal Depression Scale at baseline, 1 week, and 1 month following the intervention. Data were analyzed using SPSS version 25, with statistical significance set at *p* < 0.05.

**Results:**

At baseline, no significant differences were observed between the groups in depression or state–trait anxiety scores. Post‐intervention assessments demonstrated significant reductions in depression and anxiety symptoms in the intervention group compared with the control group at both follow‐up time points (*p* < 0.05). In addition, scores on the negotiation subscale of the Conflict Tactics Scales showed significant improvement in the intervention group 1 week and 1 month after the intervention. Levels of psychological violence reported by participants were also significantly lower in the intervention group at post‐intervention and 1‐month follow‐up (*p* < 0.05).

**Conclusion:**

Positive psychology–based interventions appear to be effective in alleviating psychological distress and strengthening conflict resolution strategies among pregnant women exposed to intimate partner violence.

**Abbreviations**
HITS: The Hurt, Insult, Threaten, Scream
IPV: intimate partner violence
STAI: state‐trait anxiety inventory

## 1 Introduction

The COVID‐19 pandemic has created unprecedented complications in every aspect of life, including social, economic, and health domains. Among these challenges, an increase in intimate partner violence has emerged as a significant issue exacerbated by the pandemic [1]. Intimate partner violence during pregnancy encompasses physical, sexual, and psychological abuse perpetrated by a current or former partner throughout pregnancy or within the first year postpartum [2, 3]. This form of violence transcends cultural, economic, and religious boundaries and affects women across diverse populations. Pregnancy and the perinatal period constitute especially sensitive phases, during which women may be more susceptible to violence due to increased physical dependency, emotional vulnerability, and socioeconomic demands [4].

Reported prevalence rates of IPV during pregnancy vary considerably across regions, ranging from 2% to 35%, although underreporting remains a substantial concern [2, 3]. In Iran, a systematic review and meta‐analysis documented that nearly half of pregnant women reported exposure to IPV [5].

Perinatal IPV has been linked to a wide spectrum of adverse mental health and obstetric outcomes, affecting both maternal and fetal well‐being [6]. These include elevated risks of preterm delivery, fetal growth restriction, miscarriage, and perinatal mortality [7]. Women exposed to IPV are also more likely to delay or inadequately utilize prenatal healthcare services [8]. Consequently, routine IPV screening is recommended during prenatal and postpartum care [7]. Psychological sequelae such as anxiety and depression frequently accompany IPV during pregnancy [9, 10].

Perinatal depression, occurring during pregnancy or shortly after childbirth, represents a major public health concern. Its prevalence has been estimated at approximately 15% in high‐income countries and up to 40% in low‐ and middle‐income settings [11, 12].

Antenatal depression is among the strongest predictors of postpartum depressive disorders [13, 14]. One study found that during the initial weeks of pregnancy, the prevalence rates of prenatal stress, anxiety, and depression were 91.86%, 15.04%, and 5.19%, respectively [15]. Similarly, an Iranian study reported the prevalence rates of depression, anxiety, and stress to be 31.7%, 32.5%, and 49.1%, respectively [16].

Interpersonal conflict plays a central role in the dynamics of intimate partner violence. Conflict arises from incompatibilities in goals, behaviors, or expectations between individuals and, when poorly managed, may escalate into violent interactions [17, 18].

Various conflict resolution methods exist, collectively referred to as conflict resolution styles. Studies have used different types of interventions aimed at reducing marital conflicts, such as couples therapy and family therapy (solution‐focused short‐term therapy) [19], the Unified Model by Feldman [20] and Forgiveness in Therapy [21].

Conflict resolution strategies—defined as the behavioral and cognitive approaches used to address disagreements—are therefore critical targets for intervention. Existing approaches, such as couples therapy, family‐based interventions, unified therapeutic models, and forgiveness‐based therapies, have demonstrated varying degrees of effectiveness [19, 20, 21].

Positive psychology offers an alternative, strength‐based framework that emphasizes the cultivation of positive emotions, adaptive coping, and personal strengths rather than focusing solely on pathology [22, 23].

Positive psychology interventions (PPIs) have shown promising effects in enhancing psychological well‐being and reducing symptoms of depression and distress across diverse populations [24]. An internet‐based interventions based on positive psychology, metacognitive therapy, and couples therapy (“Mamma Mia”) during pregnancy and after birth, reduced the risk for postpartum depression and enhanced subjective well‐being [25]. Matvienko and Samantha investigated the effects of a novel positive psychological intervention on prenatal stress and well‐being, concluding that a brief online mindfulness and gratitude intervention had the potential to reduce stress during pregnancy [26]. Pluess et al. suggested that positive life experiences during the prenatal period may serve as protective psychosocial resources, potentially buffering the negative effects of stress and adversity on both the mother and the developing fetus [27]. A recent study demonstrated that positive effects on pregnancy could act as a protective factor against postpartum depression [28]. Hyeon and Yeong reported that adopting a positive psychology‐based program can potentially prevent depression in pregnant women suffering from depression [29].

Positive psychology interventions can enhance well‐being and decrease depressive symptoms [23]. While IPV during pregnancy increased overall during the COVID‐19 pandemic, violence and related gender‐based power disparities were prominent prior to the onset of the pandemic accordingly, intervention such as psychological interventions elevating depression, anxiety, and conflict resolution among pregnant women experiencing IPV is essential [30].

Considering the high prevalence of intimate partner violence and the documented increase in its incidence during COVID‐19–related lockdowns, the issue warrants urgent attention [30, 31]. Pregnancy represents a period of heightened vulnerability, and IPV can have detrimental effects on both maternal and fetal health. Therefore, investigating psychological interventions aimed at reducing depression and anxiety symptoms and enhancing conflict resolution skills in affected populations holds significant promise.

This study was theoretically grounded in the broaden‐and‐build theory, which conceptualizes positive emotions as mechanisms that broaden cognitive and behavioral repertoires and promote the accumulation of enduring psychological and social resources necessary for resilience and well‐being [32]. Drawing on this theoretical framework, the present study aimed to assess the effectiveness of an 8‐week positive psychology–based intervention in reducing depressive and anxiety symptoms and enhancing conflict resolution strategies among pregnant women exposed to intimate partner violence during the COVID‐19 pandemic.

## 2 Methods

### 2.1 Study Design and Study Setting

This quasi‐experimental study was carried out in public health centers located in Kermanshah, a city in western Iran, between September 2021 and April 2022. Among the 28 health centers operating in the region, four centers were randomly selected to serve as recruitment sites. Participant enrollment at each center was conducted proportionally according to the number of pregnant women receiving prenatal services at that facility.

### 2.2 Participants

**Figure 1:**
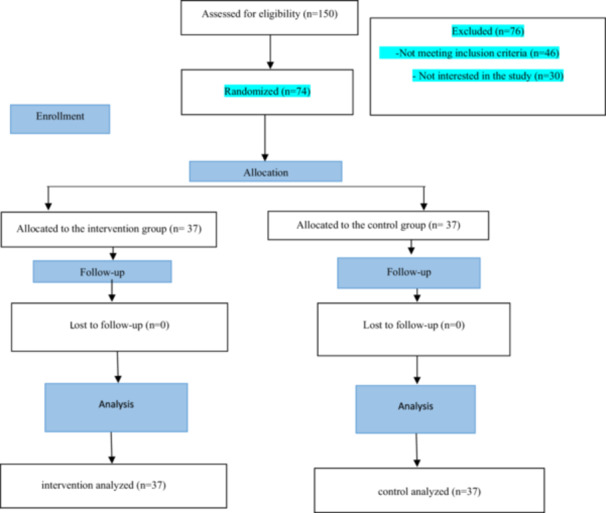
CONSORT flowchart of the study.

Initially, 150 pregnant women were screened to assess eligibility for participation. Of these, 74 women met the inclusion criteria and were enrolled in the study. Participants were randomly allocated to either the intervention group or the control group, with 37 individuals assigned to each arm. No participants withdrew during the study period, and complete outcome data were obtained from all enrolled women, resulting in a final sample size of 74 participants (Figure 1).

Identification of potential participants was based on reports from health centers and screening results obtained using the Hurt, Insult, Threaten, Scream (HITS) domestic violence questionnaire. Screening for IPV was conducted by the first author via telephone interviews using the HITS instrument.

### 2.3 Eligibility Criteria

Women were eligible for inclusion if they were between 18 and 45 years of age, married, at a gestational age of 6–30 weeks, and scored ≥ 10 on the HITS questionnaire. Additional inclusion criteria included the absence of diagnosed physical or psychological disorders and no history of alcohol or substance use. Exclusion criteria comprised unwillingness to continue participation, high‐risk pregnancy, divorce or spousal death during the study period, and absence from two or more intervention sessions.

Eligibility was confirmed by trained research personnel through standardized questionnaires and structured interviews assessing mental health status, substance use, and other eligibility conditions. Following the provision of written informed consent, eligible participants were randomly assigned to either the intervention or control group.

Participants allocated to the intervention group received a structured positive psychology program consisting of 8 weekly sessions. Each session lasted approximately 60–90 min and was delivered in small groups of three participants. Due to COVID‐19–related safety considerations, all intervention sessions were conducted remotely via videoconferencing using WhatsApp. Participant recruitment and study procedures adhered to public health guidelines, including mask use, hand hygiene, and physical distancing.

A control group was included to distinguish intervention effects from the natural progression of psychological symptoms. Participants in the control group received standard prenatal care routinely provided at health centers. This care included assessments of weight, blood pressure, fundal height, and fetal heart rate, as well as education on oral and dental health, sexual health, and screening for domestic violence, smoking, alcohol use, and substance use.

### 2.4 Intervention

In addition to routine prenatal care, participants in the intervention group received a structured positive psychology–based program consisting of 8 weekly sessions, each lasting approximately 60–90 min. The intervention protocol was developed by the third author, drawing on established positive psychology frameworks proposed by Shin and Shin [29] and Yaghoobi and Moghadam [33]. Prior to implementation, the program was pilot‐tested with five pregnant women, and subsequent refinements were made based on feedback from experts in psychology and obstetrics/gynecology. The finalized intervention was then delivered to the intervention group.

A detailed overview of session content is presented in Table 1. The intervention was conducted online using WhatsApp videoconferencing. To facilitate interaction, discussion, and peer engagement, participants were organized into small groups of three, resulting in multiple online groups accommodating a total of 37 women.

**Table 1:** Summary of positive psychology‐based intervention method [29, 33].

Session	Goals	Content
1	Exploring the positive thinking concepts and positive psychology, and their impact on pregnancy and fetal outcomes	− Instances of violence and family conflicts − Timing and method of conducting positive psychology counseling sessions − Positive psychology and the concept of positive thinking − Prerequisites for participation in the study
2	Educating the patient to introduce herself positively and to be familiar with personal strengths: how to develop and cultivate strengths for achieving happiness in life	− Question and answer on key points from the previous session − Positive self‐introduction − Introduction to personal strengths − How to develop and cultivate personal strengths − Strategies for achieving happiness in life
3	Operationalizing personal strengths: a cognitive theory and the expansion of positive emotions	Follow‐up, questioning, and answering questions regarding the previous homework assignment and the identification of strengths in the positive self‐introduction narratives. a. Review and examination of the positive self‐introduction narratives. b. Operationalizing strengths. c. Confirmation from the construct and expansion theory of positive emotions. d. How our thoughts influence our behavior and emotions.
4	Determining the value of positive emotions	– Discussion and review of the homework assignment from the previous session – Examination of participants' plans and programs for harnessing their abilities and utilizing them. a The value of positive emotions and their relationship with abilities b volunteering c be your friend d boosting self‐esteem e guidelines for practicing gratitude
5	Familiarizing with the impact of positive thinking on health and longevity, emotional discharge, and expression of emotions	Exploring gratitude memories of participants and following up on homework assignments from the previous session at the beginning of the session, inquiring about participants' perspectives on the value of positive emotions in life, and assessing their worksheets. Addressing themes such as the impact of positive thinking on health and longevity, presenting models that positive thinking has given meaning to people's lives, and providing opportunities for emotional release and expression of feelings and concerns. a Realism b things to the best of your ability c Emotional venting and expression d Writing three negative memories e Completing the happy and enjoyable life questionnaire
6	Guiding the patient in writing a forgiveness letter, negative thoughts, and strategies for their moderation	Discussing assignments from the previous session (three bad memories and the causes) at the beginning of the session and then examining and scoring the Happy and Joyful Life Questionnaire
7	Familiarizing with the miracle of gratitude: guiding the patients in writing for composing a thank‐you letter and its delivery	– Emphasizing the impact of appreciation and gratitude on memories – Teaching the participants how to write a gratitude letter to someone they have never properly thanked before, encouraging them to include specific details, and sending it to them.
8	Guiding the patients toward contentment against maximalism: How to cultivate greater satisfaction in practicing contentment	a Finding a meaning for life b Having a life philosophy c Guideline of contentment against maximalism

The intervention sessions were facilitated by the first and third authors. Group leadership was provided by a PhD‐level clinical psychologist with expertise in positive psychology (third author). The first session was conducted individually to establish rapport, collect participants' background information, and introduce the structure and objectives of the program. During this session, participants were informed that subsequent sessions would be conducted in small group formats, and the schedule for upcoming sessions was determined.

According to studies, internet‐based interventions based on positive psychology, metacognitive therapy, and couples therapy (“Mamma Mia”), reduce the risk for postpartum depression and enhance subjective well‐being [25].

Additionally, videos and worksheets were uploaded and sent via WhatsApp. Evaluations were also conducted through the application. All pregnant women used their smartphones to join the sessions.

The initial session introduced foundational concepts of positive psychology and positive thinking, with particular emphasis on their relevance to pregnancy and fetal well‐being. Participants discussed experiences related to violence and family conflict, followed by clarification of the counseling process, session timelines, and study expectations.

The second session focused on fostering positive self‐perception through structured self‐introduction exercises and identification of personal strengths. Participants were guided in recognizing individual strengths and exploring strategies to cultivate these strengths to enhance life satisfaction and well‐being.

In the third session, participants reviewed previous assignments and examined ways to apply identified strengths in daily life. Core principles of the broaden‐and‐build theory of positive emotions were introduced, highlighting the reciprocal relationships among thoughts, emotions, and behaviors.

The fourth session emphasized the role and value of positive emotions. Participants reviewed homework activities, reflected on plans for utilizing personal capabilities, and engaged in discussions on self‐compassion, self‐esteem, volunteering, and gratitude practices. Practical guidance on cultivating gratitude was also provided.

The fifth session addressed the influence of positive thinking on physical health, emotional regulation, and longevity. Participants shared gratitude‐related experiences, discussed emotional expression and release, and completed reflective exercises, including writing about negative memories and responding to the “Happy and Enjoyable Life” questionnaire.

The sixth session centered on forgiveness and cognitive regulation strategies. Participants practiced writing forgiveness letters, identified maladaptive thought patterns, and learned techniques to moderate negative cognitions. Homework assignments and questionnaire results from the previous session were reviewed and discussed.

The seventh session introduced advanced gratitude practices, emphasizing the effects of appreciation on emotional memory and psychological well‐being. Participants were instructed to write a detailed gratitude letter to an individual they had not previously acknowledged and were encouraged to deliver the letter.

The final session focused on cultivating contentment and reducing maximalist tendencies. Participants explored approaches to meaning‐making, development of a personal life philosophy, and strategies for fostering satisfaction and acceptance in daily life.

To reinforce session content, educational materials summarizing key concepts were distributed in pamphlet format via WhatsApp following each session. Upon completion of the eighth session, participants were informed that follow‐up assessments would be conducted 1 week later. Outcome questionnaires were subsequently distributed electronically via WhatsApp by a research assistant, and participants were asked to complete them accurately.

Outcome measures assessing depression, anxiety, and conflict resolution strategies were collected at baseline, 1 week after the intervention, and 1 month after the intervention. Participants in both study groups were followed longitudinally, with regular reminders provided to enhance adherence and minimize attrition. Participants were individually monitored throughout the intervention period to promote adherence and completion of assigned activities.

All questionnaire data were collected electronically during pregnancy.

### 2.5 Sample Size Calculation

To determine the sample size, an a priori power analysis was conducted prior to the implementation of the study using G*Power software (version 3.1). The calculation was based on an F‐test within a repeated measures design (Repeated Measures ANOVA; group × time interaction effect) including two groups and two measurement points (pre‐test and post‐test).

Assuming a medium effect size (*f* = 0.25), a significance level of *α* = 0.05, and a statistical power of 0.80, the minimum required sample size was estimated to be *n* = 74 participants. Ultimately, a total of *n* = 74 participants were enrolled in the study.

In the final analysis, pre‐test scores were entered into the model as a covariate to control for potential baseline differences between the groups.

### 2.6 Randomization

To select recruitment sites, Kermanshah city was geographically stratified into four areas (northwest, northeast, southwest, and southeast). A comprehensive list of health centers within each area was prepared, and one center from each region was randomly chosen. Within the selected centers, participants were recruited using quota sampling proportional to the number of registered pregnant women at each site, based on center records.

Eligible participants were assigned to either the intervention group (*n* = 37) or the control group (*n* = 37) using block randomization with a block size of four. The random allocation sequence was generated by an independent individual who had no involvement in participant recruitment, intervention delivery, outcome assessment, or data analysis. Randomization was conducted using the Sealed Envelope web‐based randomization system, resulting in the creation of 19 blocks to reach the target sample size.

Allocation concealment was ensured by assigning each participant a unique identification code generated automatically by the randomization platform. The research team remained unaware of the allocation sequence and was unable to predict group assignments. Eligibility verification and assignment of random codes were carried out by an independent third party. In addition, outcome assessments were performed by a trained assessor who was blinded to group allocation. These procedures were implemented to minimize selection and assessment bias and to strengthen the internal validity of the study.

### 2.7 Data Collection

Data were collected using a demographic and obstetric information form, the Hurt, Insult, Threaten, Scream (HITS) domestic violence checklist, the Conflict Tactics Scales (CTS‐2, victim form), the Spielberger State–Trait Anxiety Inventory, and the Edinburgh Depression Scale. All instruments were administered to participants in both the intervention and control groups at baseline, 1 week after completion of the intervention, and 1‐month post‐intervention.

Follow‐up assessments at 1 week and 1 month were selected to evaluate both immediate and short‐term effects of the eight‐session psychological intervention. These time points were chosen to enhance feasibility and reduce attrition, which is a common challenge in studies involving pregnant women exposed to intimate partner violence. Short‐term follow‐up intervals are also commonly used in pregnancy‐related psychological intervention research, allowing timely evaluation of intervention effectiveness while accounting for ethical and practical constraints associated with the perinatal period.

#### 2.7.1 The Hits Domestic Violence Checklist

The Hurt, Insult, Threaten, Scream (HITS) questionnaire is a brief screening instrument originally developed in the United States in 1998 for use in primary care settings to identify domestic violence [34]. The scale comprises four items assessing experiences of verbal and physical abuse. Responses are rated on a five‐point Likert scale, yielding total scores ranging from 4 to 20, with scores greater than 10 indicating exposure to violence.

The Persian version of the HITS questionnaire was standardized and validated in Iran by Shirzadi et al. [35] Psychometric evaluation demonstrated excellent internal consistency, with Cronbach's alpha coefficients of 0.994, 0.984, 0.977, and 0.981 for the first through fourth items, respectively. These findings indicate a high level of reliability for the Persian adaptation of the HITS scale.

#### 2.7.2 The State‐Trait Anxiety Inventory (STAI)

The State–Trait Anxiety Inventory is a widely used self‐report measure designed to assess both situational (state) and dispositional (trait) components of anxiety [36]. The instrument includes two 20‐item subscales. The State Anxiety subscale evaluates transient emotional states experienced at the time of assessment, whereas the Trait Anxiety subscale (Form Y‐2), also referred to as the hidden or covert anxiety scale, measures individuals' general and enduring anxiety tendencies.

Items on both subscales are rated using a four‐point Likert format. For the State Anxiety subscale, response options range from 1 (very low) to 4 (very high), while the Trait Anxiety subscale uses response categories from 1 (rarely) to 4 (almost always). Higher total scores on each subscale reflect greater levels of anxiety [32].

The Persian version of the STAI (Form Y) has been validated in Iran by Abdoli et al. [37] who reported satisfactory internal consistency coefficients, with Cronbach's alpha values of 0.886 for trait anxiety and 0.846 for state anxiety. Convergent validity was supported through significant correlations with the Beck Anxiety Inventory (r = 0.612 for trait anxiety and r = 0.643 for state anxiety; *p* < 0.001).

In the present study, the internal consistency of the overt (state) anxiety subscale was assessed at three time points: baseline, 1 week after the intervention, and 1‐month post‐intervention. Cronbach's alpha coefficients at these assessments were 0.599, 0.731, and 0.824, respectively. Similarly, reliability estimates for the covert (trait) anxiety subscale were 0.638, 0.730, and 0.747 at the corresponding time points. Collectively, these results indicate acceptable reliability of the anxiety measures across all assessment occasions.

#### 2.7.3 The Edinburgh Postnatal Depression Scale (EPDS)

The Edinburgh Postnatal Depression Scale, developed by Cox in 1987, is a widely used instrument designed to assess depressive symptoms over the preceding week [38]. While originally intended for postpartum women, the scale can also be applied to non‐postnatal populations and men, in which case it is referred to as the Edinburgh Depression Scale (EDS) [39] The EPDS has demonstrated clinical utility as a screening tool and is sensitive to changes in mood and affect during pregnancy and the postpartum period [40]. The scale comprises 10 items, each rated on a four‐point scale ranging from “never” to “most of the time.” Total scores are calculated by summing responses across all items, yielding a range from 0 to 30. A score of 12 or higher is generally considered indicative of clinically relevant depressive symptoms [38].

The Persian version of the EPDS has been validated by Ahmadi et al., who reported a Cronbach's alpha of approximately 0.70, with concurrent validity demonstrated through correlations with the Beck Depression Inventory (*r* = 0.44) [41]. Additionally, Amiri et al. reported positive correlations between the EPDS and the Perinatal Anxiety Screening Scale (PASS) in an Iranian population [42].

In the current study, internal consistency was evaluated at three time points: baseline, 1‐week post‐intervention, and 1 month after the intervention. Cronbach's alpha values at these assessments were 0.767, 0.771, and 0.621, respectively, indicating acceptable reliability of the instrument throughout the study period.

#### 2.7.4 The Conflict Tactics Scales (CTS‐2)

The Conflict Tactics Scales, Second Edition (CTS‐2), developed by Straus et al. in 1996, represents a revised version of the original CTS (CTS‐1) [43]. The scale has been adapted and validated for use in Iran by Panaghi et al., consisting of 52 items divided equally between the perpetrator and victim forms, with 26 items in each. The CTS‐2 encompasses three subscales: negotiation (6 items), psychological aggression (8 items), and physical assault (12 items). Total scores can range from 0 to 364. Based on total scores, conflict resolution tactics are classified as weak (0–121), moderate (121–182), or strong (> 182). Validation studies conducted by Panaghi et al. demonstrated satisfactory psychometric properties for the Iranian population, with Cronbach's alpha coefficients ranging from 0.66 to 0.86 across subscales, supporting the instrument's reliability and suitability for research and couple counseling [44].

### 2.8 Ethical Considerations

The present study was conducted following the ethical principles outlined in the declaration of Helsinki and in accordance with national regulations governing research with human participants. Prior to enrollment, all participants provided written informed consent and were assured of the confidentiality of their responses. Participation was voluntary, and individuals were informed that they could withdraw from the study at any stage without consequences. Throughout the intervention period, participants were reminded of the importance of seeking professional support if they experienced depressive symptoms.

The trial was designed and reported in line with CONSORT guidelines for non‐pharmacological interventions, promoting transparency, methodological rigor, and completeness in reporting.

Participants identified as requiring additional clinical support were referred to appropriate healthcare services. To ensure equitable care, control group participants were offered the opportunity to receive the intervention after the follow‐up period, if desired.

Ethical approval for the study was obtained from the Ethics Committee of Kermanshah University of Medical Sciences (IR. KUMS. REC.1400.250).

### 2.9 Data Analysis

Statistical analyses were performed using SPSS version 25. The Kolmogorov–Smirnov test was applied to evaluate whether the distributions of scores for the Conflict Tactics Scales, the Edinburgh Depression Scale, and the State–Trait Anxiety Inventory conformed to normality assumptions. Differences in baseline demographic characteristics between the intervention and control groups were examined using chi‐square tests for categorical variables and independent‐samples t‐tests for continuous variables.

To assess changes within each group over time (baseline, 1‐week post‐intervention, and 1‐month post‐intervention), repeated‐measures analysis of variance (ANOVA) was conducted.

Given the presence of a statistically significant difference between the two groups in the physical assault subscale of the conflict tactics scales at the pre‐test stage, pre‐test scores were entered into the analysis as a covariate to control for baseline differences between the groups.

Accordingly, a repeated measures analysis of variance (ANOVA) was performed while adjusting for the effect of pre‐test scores. A significance level of *p* < 0.05 was adopted for all statistical tests.

## 3 Results

Of the 150 pregnant women initially screened, 74 met the inclusion criteria and were enrolled in the study. Participants were randomly allocated to either the intervention group or the control group, with 37 women in each arm. No participants withdrew during the follow‐up period, resulting in a complete dataset for all enrolled individuals (Figure 1).

The results of the Kolmogorov–Smirnov test indicated that the distribution of the quantitative demographic variables, as well as the dependent variables of depression, anxiety, and conflict resolution, followed a normal distribution (*p* > 0.05).

The findings of the independent samples *t*‐test demonstrated that there were no statistically significant differences between the two groups in terms of mean age, gestational age, and spouse's age (*p* > 0.05).

Furthermore, the chi‐square test results revealed no significant differences between the intervention and control groups with respect to participants' educational level, husbands' educational level, occupation, household income, number of children, and husbands' occupation (*p* > 0.05). These findings indicate that the two groups were comparable at baseline (Table 2).

**Table 2:** Baseline demographic and personal characteristics of pregnant women in the intervention and control groups.

Variable		Intervention group *n* = 37 Mean ± SD or N (%)	Control group *n* = 37 Mean ± SD or *N* (%)	*p* value
Women's age		30.30 ± 5.05	30.30 ± 5.30	0.983*
Gestational age(week)		29.50 ± 4.90	29.80 ± 4.90	0.774*
Husband's age (years)		34.30 ± 4.50	34.50 ± 4.30	0.819*
Participants' educational level	Under diploma	9 (24.3)		15 (40.6)
	0.336**			
	Diploma	16 (43.3)		13 (35.1)
	academic	12 (32.4)		9 (24.3)
Husband's education level	Under diploma	19 (51.3)		14 (37.8)
	0.336**			
	Diploma	8 (21.7)		17 (45.9)
	academic	10 (27.0)		6 (16.3)
Occupation	House wife	31 (83.8)		31 (83.8)
	*p* > 0.99**			
	Employed	6 (16.2)		6 (16.2)
Husband's occupation	Freelancer	23 (62.2)		31 (83.8)
	Office employee	11 (29.7)		5 (13.5)
		0.126**		
	Others	3 (8.1)		1 (2.7)
Number of children	0	11 (29.8)		6 (16.2)
	0.418**			
	1	19 (51.3)		23 (62.1)
	2–3	7 (18.9)		8 (21.7)
Household income	poor	7 (18.9)		5 (13.5)
	0.419**			
	medium	24 (64.9)		29 (78.4)
	good	6 (16.2)		3 (8.1)

* Independent *t*‐test

** Chi‐square test.

As presented in Table 3, baseline comparisons using independent‐samples t‐tests revealed no significant differences between the intervention and control groups in depression, overt anxiety, or covert anxiety scores (*p* > 0.05).

**Table 3:** Mean total scores of the Edinburgh depression scale and the state‐trait anxiety inventory in the intervention and control groups at baseline, one week, and one‐month post‐intervention.

Variable/Time		Independent *t*‐test		*p*‐value*	Repeated measures		
		Intervention group	Control group				
		Mean ± standard deviation	Mean ± standard deviation		Time**	Time × group	Groups
EDS total score	baseline	13.8 ± 7.6	13.6 ± 5.1	0.861	F = 6.6 *p* = 0.002 η^2^ = 0.085	F = 6.2 *p* = 0.003 η^2^ = 0.08	F = 13 *p* = 0.001 η^2^ = 0.153
	one‐week post‐intervention	9.08 ± 6.3	13.2 ± 5.1	0.003			
	one‐month post‐intervention	8.1 ± 5.6	13.7 ± 4.8	0.001			
Overt anxiety	baseline	44.4 ± 4.6	45.1 ± 3.8	0.529	F = 2.4 *p* = 0.091 η^2^ = 0.031	F = 4.3 *p* = 0.032 η^2^ = 0.054	F = 6.2 *p* = 0.014 η^2^ = 0.076
	one‐week post‐intervention	40.3 ± 14.1	45.9 ± 2.9	0.018			
	one‐month post‐intervention	39.7 ± 14.1	45.6 ± 3.1	0.014			
Covert anxiety	baseline	45.4 ± 5.8	46.5 ± 3.5	0.293	F = 4.9 *p* = 0.022 η^2^ = 0.062	F = 4.1 *p* = 0.038 η^2^ = 0.051	F = 9.5 *p* = 0.003 η^2^ = 0.112
	one week post‐intervention	39. 8 ± 14.2	46.2 ± 3.4	0.007			
	one‐month post‐intervention	39.6 ± 13.8	46.2 ± 3.08	0.004			

* Independent *t*‐test.

** Repeated measure test.

The results of the repeated measures analysis of variance demonstrated a statistically significant difference in the changes in mean depression scores among pregnant women exposed to domestic violence (*p* < 0.05). A significant interaction effect was observed between time and group. Therefore, the combined and interactive effect of the two variables (Time × Group) significantly influenced the mean depression scores of pregnant women exposed to domestic violence (*p* < 0.05). Furthermore, a statistically significant difference was found between the intervention and control groups in terms of mean depression scores (*p* < 0.05). The effect size indicated that 15.3% of the variance in depression scores in the intervention group could be attributed to the positive psychology–based intervention.

Regarding state anxiety, the findings showed no statistically significant difference in the changes in mean state anxiety scores among pregnant women exposed to domestic violence (*p* > 0.05). However, a significant interaction effect between time and group was observed. Thus, the Time × Group interaction had a statistically significant impact on the mean state anxiety scores of pregnant women exposed to domestic violence (*p* < 0.05). A statistically significant difference was also identified between the intervention and control groups in mean state anxiety scores (*p* < 0.05). The calculated effect size revealed that 7.6% of the variance in state anxiety scores in the intervention group was attributable to the positive psychology–based counseling.

With respect to trait anxiety, the results indicated a statistically significant difference in the changes in mean trait anxiety scores among pregnant women exposed to domestic violence (*p* < 0.05). A significant interaction effect between time and group was also observed. Accordingly, the Time × Group interaction significantly affected the mean trait anxiety scores of pregnant women exposed to domestic violence (*p* < 0.05). A statistically significant difference was found between the intervention and control groups in mean trait anxiety scores (*p* < 0.05). The effect size showed that 11.2% of the variance in trait anxiety scores in the intervention group could be explained by participation in positive psychology–based counseling (Table 3).

According to Table 4, the results of the independent samples *t*‐test indicated that there was no statistically significant difference between the intervention and control groups in the mean score of the negotiation subscale prior to the intervention (*p* > 0.05). Similarly, no statistically significant difference was observed between the two groups in the mean score of the psychological violence subscale before the intervention (*p* > 0.05). However, a statistically significant difference was found between the groups in the mean score of the physical assault subscale at baseline (*p* < 0.05).

**Table 4:** The mean total score of the subscales of conflict tactics scales in the positive psychology intervention and control groups at baseline, one‐ week post‐intervention, and one‐month post‐intervention.

Variable/Time			Independent *t*‐test		*p*‐value*	Time**	Time × group	Groups
			Intervention group	Control group				
			Mean ± standard deviation	Mean ± standard deviation				
victim form	Negotiation	baseline	15 ± 10	14.8 ± 9.2	0.926	F = 41.7 *p* = 0.001 η^2^ = 0.354	F = 3.2 *p* = 0.001 η^2^ = 0.042	F = 6.6 *p* = 0.012 η^2^ = 0.080
		one‐ week post‐intervention	22.1 ± 9.8	27.5 ± 7.2	0.007			
		one‐month post‐intervention	22.6 ± 10.2	28.3 ± 7.2	0.006			
	The psychological violence	baseline	19 ± 14.6	20.3 ± 8.7	0.633	F = 7.2 *p* = 0.001 η^2^ = 0.087	F = 2.9 *p* = 0.057 η^2^ = 0.037	F = 9.1 *p* = 0.003 η^2^ = 0.107
		one‐ week post‐intervention	9.3 ± 10.1	18.2 ± 8.7	0.001			
		one‐month post‐intervention	14.6 ± 14.1	20.7 ± 9.8	0.030			
	The physical assault	baseline	19.1 ± 21.3	11.3 ± 11.4	0.049	F = 0.1.3 *p* = 0.256 η^2^ = 0.018	F = 0.550 *p* = 0.578 η^2^ = 0.007	F = 3.8 *p* = 0.053 η^2^ = 0.049
		One‐ week post‐intervention	13.3 ± 19	10.1 ± 10.4	0.356			
		one‐month post‐intervention	15 ± 20.2	10.3 ± 10.3	0.199			

* Independent *t*‐test.

** Repeated measure test.

The repeated measures analysis of variance demonstrated that the mean negotiation score changed significantly over time (*p* < 0.05). The interaction effect between group and time was also statistically significant (*p* < 0.05). Therefore, the combined and interactive effect of Time × Group significantly influenced the mean negotiation scores among pregnant women exposed to domestic violence (*p* < 0.05), and the difference between the intervention and control groups was statistically significant (*p* < 0.05). The effect size indicated that 8% of the variance in the negotiation subscale in the intervention group was attributable to participation in positive psychology–based intervention.

Furthermore, the findings from the repeated measures ANOVA showed that the mean score of psychological violence changed significantly over time (*p* < 0.05). However, the interaction effect between group and time was not statistically significant (*p* > 0.05). Despite this, the difference between the two groups was statistically significant (*p* < 0.05). The effect size revealed that 10.7% of the variance in psychological violence scores in the intervention group could be explained by the positive psychology–based intervention.

In addition, the results of ANOVA indicated that the mean score of physical assault did not change significantly over time (*p* > 0.05), and the difference between the intervention and control groups was not statistically significant (*p* > 0.05) (Table 4).

## 4 Discussion

The findings of this study indicate that an 8‐week positive psychology intervention effectively reduced symptoms of anxiety and depression in pregnant women exposed to intimate partner violence, with improvements maintained at 1‐month post‐intervention.

These results align with prior research suggesting that positive psychology–based programs, including brief online mindfulness and gratitude exercises or integrated interventions combining metacognitive and couples therapy approaches, can enhance mental well‐being and buffer stress during pregnancy [25, 26, 27, 28, 29, 45, 46, 47]. Positive psychological interventions focus on cultivating positive emotions, identifying personal strengths, and promoting adaptive coping strategies, which collectively contribute to improved emotion regulation and reduced depressive symptoms [48, 49, 50, 51].

In addition to alleviating anxiety and depression, the intervention significantly enhanced participants' use of constructive conflict resolution strategies, as reflected in increased scores on the negotiation subscale of the Conflict Tactics Scales. This subscale captures efforts to resolve disagreements through cognitive and emotional negotiation, prioritizing mutual care, offering compromises, and articulating reasons for disagreement. Such strategies are consistent with conflict theory, which conceptualizes conflict management along a continuum from aggressive to nonviolent approaches. By emphasizing negotiation and adaptive emotional responses, positive psychology interventions may prevent escalation of marital conflicts and promote emotional closeness and mutual support within couples [52, 53, 54].

The intervention also resulted in reduced scores on the psychological violence subscale, suggesting a decrease in verbal and emotional abusive behaviors such as shouting, threats, or accusations of infidelity. These findings underscore the potential of positive psychology–based programs to mitigate relational stressors that often co‐occur with other forms of violence, including physical and sexual abuse [55, 56, 57]. By supporting participants in identifying constructive responses to challenging situations, these interventions may help prevent the cycle of abuse and improve overall marital functioning.

Overall, positive psychology provides a framework for helping women navigate both the positive and negative aspects of their lives. By fostering awareness of personal strengths, promoting adaptive coping, and reframing negative experiences, participants can enhance self‐assessment, psychological growth, and marital satisfaction, including improved communication and conflict resolution skills. These outcomes are particularly relevant for women exposed to intimate partner violence, as the intervention encourages them to recognize positive elements within adverse circumstances and develop strategies to transform challenges into opportunities for personal development [58].

### 4.1 Limitations and Strengths

Although this study was conducted during a pandemic, its findings are highly relevant to non‐pandemic contexts as well. Intimate partner violence and its psychological consequences—such as depression, anxiety, and impaired conflict resolution—remain major public health concerns for pregnant women regardless of global crises. The positive psychology intervention examined in this study targets fundamental psychological processes rather than stressors unique to the pandemic, suggesting its potential utility for integration into routine antenatal care and community mental health services. These low‐intensity, scalable interventions may serve as sustainable strategies for enhancing maternal mental well‐being and relational functioning in standard clinical settings.

However, some limitations warrant consideration. The relatively small sample size restricts the generalizability of the findings and underscores the need for future studies with larger cohorts. Follow‐up was limited to 1 week and 1‐month post‐intervention, chosen to balance participant retention with feasibility within the timeframe of pregnancy. While these intervals are common in pregnancy‐focused psychological intervention research, longer‐term follow‐up would provide valuable insight into the durability of the observed effects.

The remote delivery of the intervention also represents a limitation, although this was partially mitigated through video conferencing sessions, supplementary worksheets, and informational pamphlets. Finally, the absence of spousal participation due to socio‐cultural constraints may limit the comprehensiveness of the intervention and its applicability in contexts where partner involvement is feasible.

Several strengths of the study should be noted. Follow‐up assessments were conducted at two post‐intervention time points, allowing for evaluation of both immediate and short‐term effects. In addition, all instruments employed were validated and standardized for the Iranian population, enhancing the reliability and cultural relevance of the measurements.

## 5 Conclusion

The present study demonstrates that positive psychology–based interventions can alleviate symptoms of anxiety and depression while improving conflict resolution strategies in pregnant women experiencing intimate partner violence. These findings highlight the potential value of integrating low‐intensity, psychologically focused programs into prenatal care to support maternal mental health and relational functioning. Future research should explore the long‐term sustainability of these effects and consider the inclusion of partners in the intervention to assess its impact on dyadic conflict resolution and overall marital dynamics.

## Author Contributions

**Sohaila Ordikhani:** resources, methodology, writing – original draft. **Sousan Heydarpour:** conceptualization, methodology, project administration, supervision, writing, reviewing, and editing the original draft. **Nasrin Jaberghaderi:** validation, methodology. **Nader Salari:** methodology, formal analysis, validation, data curation.

## Ethics Statement

The participants signed an informed written consent. The study was approved by the Ethics Committee of Kermanshah University of Medical Sciences (IR.KUMS.REC.1400.250). All guidelines and regulations outlined in the Declaration of Helsinki were adhered to during the study's implementation.

## Conflicts of Interest

The authors declare no conflicts of interest.

## Transparency Statement

The lead author Sousan Heydarpour affirms that this manuscript is an honest, accurate, and transparent account of the study being reported; that no important aspects of the study have been omitted; and that any discrepancies from the study as planned (and, if relevant, registered) have been explained.

## Data Availability

The datasets used and/or analyzed during the current study are available from the corresponding author upon reasonable request.
